# Color doppler blood flow analysis in circumscribed choroidal hemangioma

**DOI:** 10.5935/0004-2749.2024-0167

**Published:** 2024-12-26

**Authors:** Lídia Guedes Bezerra, Maira Saad de Avila Morales, Maria Helena Mandello Carvalhaes Ramos, Melina Correia Morales, Norma Allemann

**Affiliations:** 1 Department of Ophthalmology & Visual Sciences, Escola Paulista de Medicina, Universidade Federal de São Paulo, São Paulo, SP, Brazil

**Keywords:** Ultrasonography, doppler, collor, Choroidal neoplasms, Hemangioma

## Abstract

**Purpose:**

To evaluate if color Doppler can detect internal blood flow in circumscribed choroidal hemangioma.

**Methods:**

This cross-sectional study examined seven eyes of seven participants with circumscribed choroidal hemangiomas, with or without prior treatment. B-scan ultrasound and color Doppler were used to assess the dimensions, topographical distribution, and internal blood flow of the affected eyes.

**Results:**

The sample included seven patients (five female) with a median age of 61 (62.29 ± 13.83) years. There were seven eyes with circumscribed choroidal hemangiomas in the patient sample. Color Doppler detected internal vascular flow in all cases (100%). The lesions had an average diameter/thickness ratio of >2 mm and an average thickness of <5 mm and were predominantly located superiorly and supero-temporally.

**Conclusion:**

Internal blood flow was detected in circumscribed choroidal hemangiomas using color Doppler. Detection was unaffected by the patient’s treatment status.

## INTRODUCTION

Circumscribed choroidal hemangiomas (CCHs) are rare benign primary intraocular vascular tumors of undefined etiopathogenesis^(1^,^2^,^3)^. They are congenital, vascular, and hamartomatous and are histopathologically classified as capillary, cavernous, or mixed based on the type of vessels within the lesion^(1)^.

Fundus biomicroscopy and indirect ophthalmoscopy can detect CCH tumors, which appear as orange-red masses in the subretinal space, are slightly elevated, and have indistinct borders. Yet, the clinical diagnosis of CCH is not always straightforward, and diagnostic errors can be caused by a non-specialist with limited experience or by secondary alterations resulting from the chronicity of the lesion^(4)^.

Therefore, ancillary tests may be used to facilitate the diagnosis of CCH. An ultrasound pattern specific to CCH has been described. On ultrasound with a focused 10-MHz B-scan transducer, CCH appears as a domeshaped acoustically solid lesion. They are occasionally mushroom-shaped^(5^,^6)^. On standardized A-mode scans, CCHs exhibit high internal reflectivity of 50-100% due to vascular channels within the tumor that interfere with the ultrasound beam^(7)^. While these features provide a useful means of distinguishing CCH from other choroidal lesions, internal vascularization cannot be detected using this approach. Nevertheless, it is expected that advances in technology and the improved resolution of new ocular ultrasound devices will enable the detection of internal vascular flow during kinetic examinations.

Verbeek et al.^(4)^ conducted a retrospective study of 40 patients with an ultrasound diagnosis of CCH and found uniform ultrasound characteristics among these lesions, confirming the diagnostic reliability of the method. However, a kinetic examination found no evidence of internal vascularization in the ultrasound characteristics of these lesions, contradicting the vascular nature of CCH tumors.

Color Doppler techniques do not require clear refractive media or injectable contrast agents. They are widely used to study tumor vasculature throughout the human body^(8^,^9^,^10^,^11^,^12)^. Currently, color Doppler ultrasound is used in cardiology^(13)^, cerebrovascular diseases^(14)^, vascular studies of the genitourinary system, peripheral arteriovenous diseases, and neonatal intracranial vascular studies^(15)^. However, there are few reports in the literature on the use of color Doppler in the study of the eye and orbit.

Lieb et al.^(16)^ used color Doppler to study 44 patients with intraocular lesions, four of whom had CCH. They found that, in this category of lesions, the maximum systolic and diastolic intratumoral velocities are very high. This is consistent with the pathological characteristics of these tumors.

The use of color Doppler in the workup of intraocular tumors facilitates diagnosis and enables noninvasive followup and evaluation of the therapeutic response to treatment. Advantages of this method include its noninvasiveness, safety, and repeatability^(17)^.

In patients with intraocular tumors, the detection of vascularization can aid ultrasound diagnosis^(16^,^18^,^19)^. Using B-mode ultrasound, internal vascularization is difficult to detect, and blood flow cannot be quantified^(20)^.

Therefore, this study aimed to evaluate the use of color Doppler ultrasound to detect vascular flow within the CCH.

## METHODS

This was a cross-sectional study of seven eyes of seven patients diagnosed with CCH, with or without previous treatment. The study was conducted in accordance with the tenets of the 2013 revision of the Declaration of Helsinki and was approved by our institution’s ethics committee (approval no. 52248121.9.0000.5505).

Eyes with CCH underwent B-scan and A-scan ocular ultrasound examinations (AVISO10-MHz focused transducer, Quantel Medical) and color Doppler ultrasound (Mylab Esaote 7.5-15-MHz linear transducer) using a transpalpebral approach. Conductive ultrasound gel was applied to the eyelid, avoiding contact with the eye. Longitudinal and transverse ultrasound sections of each lesion were obtained to determine their dimensions, evaluate their internal architecture, and perform Doppler blood flow analysis.

## RESULTS

The current study included seven eyes of seven participants. The mean age was 62.29 (±13.83) years (median, 61; range, 39-80 years), and the majority were female (71.43%). Lesions were more common in the left eye (71.43%), with greater involvement of the superior (42.86%) and superior temporal (42.86%) quadrants. They were mainly located in the posterior pole (85.71%). The sample consisted of four cases of primary CCH and three cases who had previously received antiangiogenic treatment with intravitreal bevacizumab (≥3 years before the examination).

**Figure 1:**
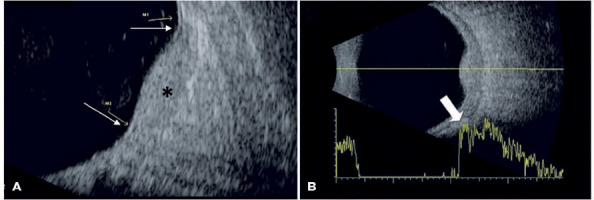
10 MHz ultrasound of a circumscribed choroidal hemangioma (*). (A) A longitudinal section from a B-scan demonstrating perilesional retinal detachment (thin arrows). The choroidal lesion is dome-shaped and characterized by homogeneous architecture and high reflectivity (*); (B) A cross-section from A and B scans with high internal reflectivity shown in the echo intensity graph (thick arrow)

**Figure 2:**
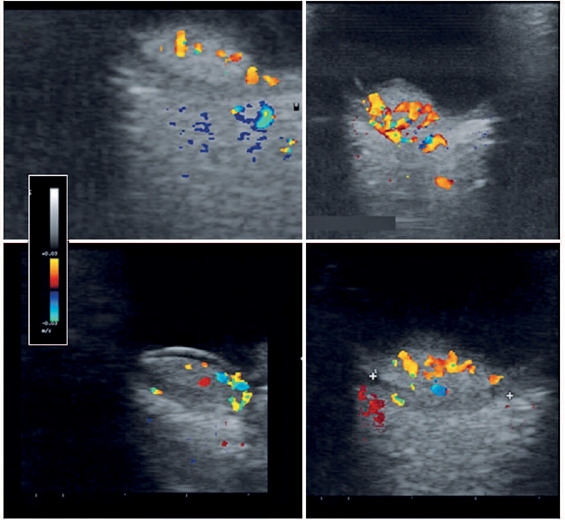
Color Doppler images (7.5–15-MHz linear transducer) from four patients with circumscribed choroidal hemangioma. (A–D) Elevated dome-shaped choroidal lesions with homogeneous architecture and color-coded intralesional blood flow.

On ultrasound, the serous retinal detachment characteristic of choroidal lesions was detected in three (42.86%) cases. The lesions showed homogeneous internal architecture (100%) and high reflectivity (85.71%) on ultrasound (Figure 1). Table 1 summarizes the clinical and ultrasound characteristics of the study sample. Table 2 summarizes the ultrasound examination measurements. Our analysis of intralesional vascular flow using color Doppler showed prominent internal vascularization in all CCHs in the sample (Figure 2).

**Table 1:** Clinical and ultrasound characteristics of patients with choroidal hemangiomas and their eyes

Variable	n (%)
Sex	
Female	5 (71.43)
Male	2 (28.57)
Laterality	
Right eye	2 (28.57)
Left eye	5 (71.43)
Quadrant	
Higher	3 (42.86)
Inferior temporal	1 (14.29)
Superior temporal	3 (42.86)
Location	
Equator	1 (14.29)
Posterior pole	6 (85.71)
Previous antiangiogenic treatment	
Yes	3 (42.86)
No	4 (57.14)
Serous retinal detachment	
Yes	3 (42.86)
No	4 (57.14)
Architecture	
Homogeneous	7(100.00)
Heterogeneous	0 (00.00)
Reflectivity	
Medium	1 (14.29)
High	6 (85.71)
Total	7 (100.00)

**Table 2:** The dimensions obtained by ultrasound examination of the circumscribed choroidal hemangiomas and axial lengths of the eyes of our cohort

	Mean	Median	Interval	P25	P75
Longitudinal diameter (mm)	9.62 ± 1.64	9.65	6.72–11.40	8.75	10.95
Circumferential diameter (mm)	9.08 ± 1.02	9.26	7.52–10.04	8.00	10.00
Thickness (mm)	4.19 ± 0.39	4.34	3.66–4.72	3.81	4.49
Longitudinal diameter/ thickness	2.29 ± 0.25	2.30	1.84–2.63	2.15	2.52
Circumferential diameter/ thickness	2.17 ± 0.23	2.05	1.96–2.63	2.03	2.27
Axial diameter of the eye (mm)	22.50 ± 1.03	22.46	21.22–23.72	21.29	23.64

## DISCUSSION

In the present study, the mean age of our cohort was 62.29 (±13.83) years (median, 61; range, 39-80 years), which is consistent with the median age of 58 years at diagnosis found in a multicenter study of 113 patients with CCH^(21)^.

We analyzed the characteristics of CCH on A-scan and B-scan ultrasound, including internal architecture, ultrasonographic pattern, diameter-thickness ratio, location, and thickness. We found a mean diameter-thickness ratio >2 and a mean thickness of 4.19 (±0.39) mm, with a range of 3.44-4.72 mm. The most common locations were superior and supero-temporal. This is consistent with the parameters obtained by Krohn et al.^(21)^, in whose sample none of the tumors had a diameter-thickness ratio of ≤2, and all had a thickness <5 mm. Similar thickness values were reported by Witschel et al.^(1)^ in a clinicopathological study of 71 patients with CCH, in which the average lesion thickness was <6 mm.

The dome shape in B-mode ultrasonography and the high reflectivity in nonstandardized A-mode ultrasonography seen in the present sample coincide with the findings of Shields et al.^(5)^, whose sample of 198 patients with CCH also showed high internal reflectivity in nonstandardized A-mode. Only two lesions were not dome-shaped in B-mode.

Although focal choroidal excavation is thought to be important for differentiating CCH from other choroidal lesions, we found it in none of our patients on ultrasound.

Our qualitative analysis of data from color Doppler ultrasounds revealed the internal vascular flow in all patients. This confirmed the vascular nature of the tumor was in accord with the findings of Lieb et al.^(16)^ in a color flow Doppler ultrasound study of four patients with CCH.

The present study used color Doppler ultrasound to successfully demonstrate the internal vascular flow of CCH lesions. This was evidenced in both untreated patients and those who had previously received antiangiogenic treatment. Our small sample size was a limitation of this study but it does not hinder the validity of our findings. The consistent detection of internal vascular flow using color Doppler in all seven patients supports our conclusions and contributes valuable insights into the diagnosis and management of CCH. Further studies with larger samples may be needed to strengthen these results, but the current findings remain relevant and significant.

In conclusion, the ancillary study of CCH with color Doppler allows the detection of intralesional blood flow, facilitating differential diagnosis of the tumor and allowing for intrapatient comparisons over time during followup.

Guedes Bezerra; Norma Allemann. **Obtaining funding:** not applicable. **Supervision of administrative, technical, or material support:** Norma Allemann. **Research group leadership:** Norma Allemann.
